# Electromyographic Analysis of the Hip Extension Pattern in Visually Impaired Athletes

**DOI:** 10.1515/hukin-2015-0091

**Published:** 2015-01-12

**Authors:** Tomasz Halski, Piotr Żmijewski, Paweł Cięszczyk, Barbara Nowak, Kuba Ptaszkowski, Lucyna Slupska, Robert Dymarek, Jakub Taradaj

**Affiliations:** 1 Institute of Physiotherapy, Public Higher Medical Professional School in Opole, Poland; 2 Department of Physiology, Institute of Sport in Warsaw, Poland; 3 Department of Physical Culture and Health Promotion, University of Szczecin, Poland; 4 Department of Team Sport Games, Academy School of Physical Education in Katowice, Poland; 5 Department of Physiotherapy, University of Medicine in Wroclaw, Poland; 6 Department of Nervous System Diseases, University of Medicine in Wroclaw, Poland; 7 Department of Physiotherapy Basics, Academy School of Physical Education in Katowice, Poland

**Keywords:** visually impaired athletes, movement pattern, hip extension, muscle recruitment time, surface electromyography

## Abstract

The objective of the study was to determine the order of muscle recruitment during the active hip joint extension in particular positions in young visually impaired athletes. The average recruitment time (ART) of the gluteus maximus (GM) and the hamstring muscle group (HMG) was assessed by the means of surface electromyography (sEMG). The sequence of muscle recruitment in the female and male group was also taken into consideration. This study followed a prospective, cross – sectional, randomised design, where 76 visually impaired athletes between the age of 18–25 years were enrolled into the research and selected on chosen inclusion and exclusion criteria. Finally, 64 young subjects (32 men and 32 women) were included in the study (age: 21.1 ± 1.05 years; body mass: 68.4 ± 12.4 kg; body height: 1.74 ± 0.09 m; BMI: 22.20 ± 2.25 kg/m2). All subjects were analysed for the ART of the GM and HMG during the active hip extension performed in two different positions, as well as resting and functional sEMG activity of each muscle. Between gender differences were comprised and the correlations between the ART of the GM and HMG with their functional sEMG activity during hip extension in both positions were shown. No significant differences between the ART of the GM and HMG were found (p>0.05). Furthermore, there was no significant difference of ART among both tested positions, as well in male as female subjects (p>0.05).

## Introduction

Movement or motor patterns (MP) are applied in numerous methods of modern physiotherapy as an element of functional diagnostics (Bruno, 2011; Janda, 1991; Janda et al., 1996). The MP are also used in therapeutic procedures (i.e. manual therapy, PNF method) (Lewis and Ferris, 2011; Lewit, 2010; Murphy et al., 2006).

Lewit (2010), author of an international handbook of manual medicine, along with Janda (1991) devote particular attention to the issue of MP (stereotypic movements). They argue that in the ideal MP the movement becomes economical due to the lowest energy expenditure. Unconditional and conditional reflexes shape the MP throughout the entire life. In ontogeny conditional reflexes become stronger as a result of higher activity and control of the cerebral cortex. Therefore, MP may show inter–individual differences. MP shaped in the course of human development through increasing body mass, the type of work, external environment or age is called a dynamic movement pattern (DMP) or an individual movement pattern (IMP). Though, MP frequently becomes incorrect (Bruno et al., 2008).

Janda et al. (1996) presented numerous research on this issue in which they focused on a range of elements distorting stereotypic movements. They covered among others, incorrect muscle work that pathologically influenced the central neurological control of movement. Long–term pain, chronic fatigue and defective body posture are also the factors that affect the development of MP.

A typical sequence of a hip extensor recruitment pattern can be modified under the influence of pathological situations, e.g. chronic pain syndromes, musculoskeletal dysfunctions and low–back disorders (Arab et al., 2011; Fabian et al., 2005; Hossain and Nokes, 2005; Vogt et al., 2003). Janda (1991) proved that incorrect MP may be the result of locked joints. Thus, a therapeutic method in the movement disorder should consist of the improvement of an incorrect pattern.

One of the patterns proposed by Lewit (2003) was a pattern of hip joint extension which he defined in the position of prone leg extension that was lower extremity extension while lying forward. The author noticed that the muscles which initiated this movement belonged to the hamstring muscle group (HMG), followed by the gluteus maximus (GM) and finally, the erector spinae (ES).

An MP assessment of hip joint extension appears to be interesting in respect to sport rehabilitation of athletes (especially paralympic subjects with complex motor skills compared to healthy ones) and patients with hip joint degenerative changes, after total hip arthroplasty or orthopaedic trauma and those with rheumatic diseases in the region of the pelvic girdle and lower extremity (Tateuchi et al., 2013). Early diagnostics by means of surface electromyography (sEMG) may play an important role in defining the sequence of muscle activation and the improvement of MP. The application of sEMG seems to be an efficient method of muscle activity assessment as it allows for an objective, quantitative and documented analysis of both resting and functional measurements (Buckthorpe et al., 2013; Morrissey et al., 2012; Park et al., 2013; Pierce et al., 2011).

The aim of the study was to determine the order of muscle recruitment during the active hip joint extension in particular positions in visually impaired athletes. The primary study endpoints were the measurement of the average recruitment time (ART) required by the GM and HMG to perform a movement of hip joint extension and the assessment of the difference in activation time between the GM and HMG in two positions; as well as the comparison of resting and functional sEMG activity of the GM and HMG. The secondary study endpoint was the assessment of the correlations between the ART of the GM and HMG with their functional sEMG activity during hip extension in both positions. Another secondary study endpoint was the comparison of the ART results between female and male subjects in two positions.

## Material and Methods

### Subjects

A group of 76 visually impaired athletes between the age of 18–25 years were enrolled in the research and submitted to the qualification procedure based on chosen inclusion and exclusion criteria. Subjects with the following conditions were excluded from the study: lower limb injuries (n=1), lower back pain (n=2), a painful hip during testing (n=2), decreased muscle elasticity (n=1), scoliosis of the spine (n=3), a pathological pelvis position (n=3), contraindications for sEMG measurement (n=1). Inclusion criteria comprised the following: lack of lower limb injuries and lower back pain within the past year, normal range of motion of the hip joint (goniometer), correct muscle elasticity and hip joint function during testing (Mennell Sing; Thomas Grip; Patrick test; Ober’s test; Fingertip test; Ely’s test; Three–Phase Hyperextension test; Hip Extension test) (Buckup, 2008), lack of dysfunctions of the spine and sacroiliac joint (Spine test; Standing Flexion test) (Buckup, 2008), a normal pelvis position and a correct pelvic tilt (inclinometer), symmetrical circumferences and lengths of lower extremities (centimeter tape, Derbolowsky Sign) (Buckup, 2008), lack of contraindications for sEMG measurement, a signed consent form of participation.

Finally, 64 subjects – 32 swimmers, 8 athletes in triple and long jumps, 10 runners, 14 goalballers (32 men and 32 women), age of 21.1 ± 1.05 years, body mass of 68.4 ± 12.4 kg, body height of 1.74 ± 0.09 m, and the body mass index (BMI) of 22.20 ± 2.25 kg/m^2^, participated in the study and were randomly qualified for the analysis. There were 32 females (age: 20.6 ± 1.04 years; BMI: 20.07 ± 1.67 kg/m^2^) and 32 males (age: 21.6 ± 0.78 years; BMI: 23.8 ± 1.59 kg/m^2^). Both studied groups were homogenous with regard to all characteristics (*p*>0.05). All subjects presented right–leg dominance, defined according to which leg they used for kicking. The lower extremity subjected to measurement was chosen randomly as follows: 36 right and 28 left lower extremities were tested. None of the subjects reported any present pathologies of the musculoskeletal, nervous or cardio–respiratory system at the time of the study.

This study followed a prospective, cross–sectional design. Before inclusion, informed consent in accordance with institutional ethical standards of the Ethics Committee on human experimentation was obtained from each subject (no. KB/01/10/2013 by Public Higher Medical Professional School in Opole, Poland).

### Measurements

The measurement was performed by a dual–channel sEMG NeuroTrac ETS^®^ device integrated with computer software for digital analysis and report creation (Verity Medical Ltd., United Kingdom). This device is characterised by an amplitude range of 0.2 – 2000 μV root mean square (RMS) continuous in the frequency band of 2 – 100 Hz and a pulse width from 50 to 450 μS for recording signals generated by muscles. Device sensitivity was established at the level of 0.1 μV (4% accuracy; readings +/− 0.3 mV at 200 Hz), with a selectable bandpass filter (3 db bandwidth) and a 50 Hz notch filter (33 dbs; 0.1% accuracy). The analogue signal recorded by the sEMG electrodes was amplified, filtered and subsequently transformed into a digital signal. The measurement of muscle recruitment time and their action potentials was performed in the same conditions (electrode attachment points, subjects’ positioning, and measurement site).

Before the application of electrodes, the skin was prepared with 70% alcohol to reduce skin impedance and also shaved if necessary. To amplify the sEMG signal, bipolar, self–adhesive, and oblong 50×50 mm active electrodes with hypoallergenic gel were used. All electrodes were placed in pairs with a distance of 1.5–2.0 cm from each other and parallel to the muscle fibers over the centre part of the GM and HMG muscles’ bellies according to the SENIAM and ISEK recommendations (Hermens et al., 2000). Electrodes placement to register sEMG activation were as follow: for the GM – at the midpoint of a line running from S_2_ vertebra to the greater trochanter; and for the HMG – laterally on the mid distance between gluteal and popliteal fold (Arab et al., 2008). The single monopolar, self–adhesive, and round 30 mm reference electrode was placed on the fibular head on the same side. All sEMG electrodes were not removed and placed at the same stable locations during the measurements in both tested positions.

Functional sEMG activity of the GM and HMG was recorded in real–time to analyse the order of the muscle activation pattern. Recruitment time was considered as the necessary time to attain 75% value of the average muscle activation during hip extension in two positions. The mean onset of muscle activity for each set was calculated from the five trials within that set in both positions separately. Mean values of resting and functional GM and HMG muscles’ bioelectrical activity were given according to the RMS algorithm (μV).

### Procedures

At the beginning, all subjects were placed for 10 min in a comfortable and safe supine position, aimed at postural adaptation. First of all, measurements of resting sEMG activity in an appropriate starting position (Position 1 and Position 2) were performed. In the next stage, active hip extension in two positions (Position 1 and Position 2) was performed, until the lower edge of the patella was raised more than 15 cm from the starting position. Subjects were instructed to execute hip extension at their natural speed, performing five trials of each movement from the starting position with 5 s rest between every trial (Takasaki et al., 2009). Subjects were given a 5 min rest period between the two positions. In both testing positions, pelvis stabilization was provided to eliminate uncontrolled movements of the body, especially of the sacroiliac joint and the lumbar spine (Caneiro et al., 2010; Hashemirad et al., 2009).

**Figure 1:**
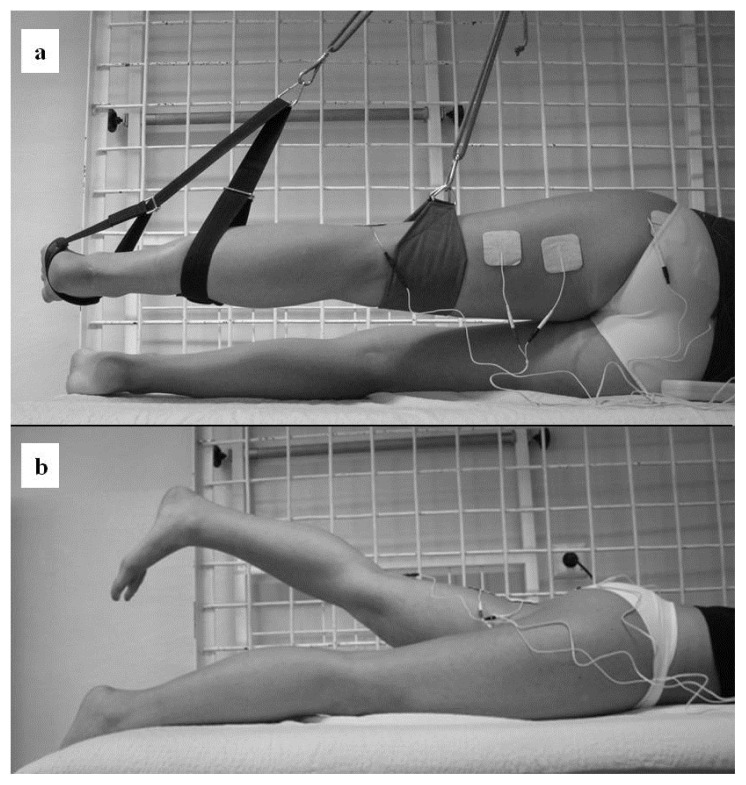
The first (a) and the second (b) testing position during hip extension with the sEMG electrodes placement over the GM and HMG

1. First position – the subjects were asked to lie lateral on a therapeutic table with pelvis stabilization, the tested lower extremity at a neutral position suspended in the Universal Exercise Unit (UEU) in accordance with kinesiotherapy principles to eliminate the force of gravity and reduce the weight of the limb to ensure facilitation conditions during testing (Position 1, Figure 1).
2. Second position – the subjects were asked to lie prone on the therapeutic table with pelvis stabilization, feet shoulder–width apart, arms at their sides and head in line in a standardized test position for prone hip extension (PHE) testing (Position 2, Figure 1).

### Statistical analysis

STATISTICA 10 software (StatSoft Company Inc. USA) was used for statistical data analysis. Arithmetic means, standard deviations and ranges of variation were calculated for measured variables. All analysed variables were checked using the Shapiro–Wilk test to establish the type of distribution. The t-test was applied for independent samples to compare muscle recruitment time, as well as average resting and functional sEMG activity. In addition, Pearson correlation was calculated to show the relationship between the variables. Statistical significance was set at *p*<0.05.

## Results

### Average sEMG activity and ART of the GM and HMG

There were strong significant differences in all obtained results in mean values of resting and functional sEMG activity of the GM and HMG in both tested positions amongst all subjects (p<0.05) (Table 1).

In Position 1 the HMG was recruited on average time in 1.45 s (range 0.1 – 1.9 ± 0.45 s), whereas the GM in 1.50 s (range 0.3 – 1.9 ± 0.33 s); in Position 2 the ART amounted to 1.37 s (range 1.0 – 1.8 ± 0.21 s) and 1.40 (range 1.0 – 1.9 ± 0.18 s), respectively. Differences between the ART of the GM in respect to the HMG were statistically non-significant (Position 1, p=0.4163; Position 2, p=0.4125). We observed a statistically significant difference in ART of the GM between Position 1 and Position 2 (p=0.0275) (Table 1).

### Correlations of ART with functional sEMG activity of the GM and HMG

**Figure 2:**
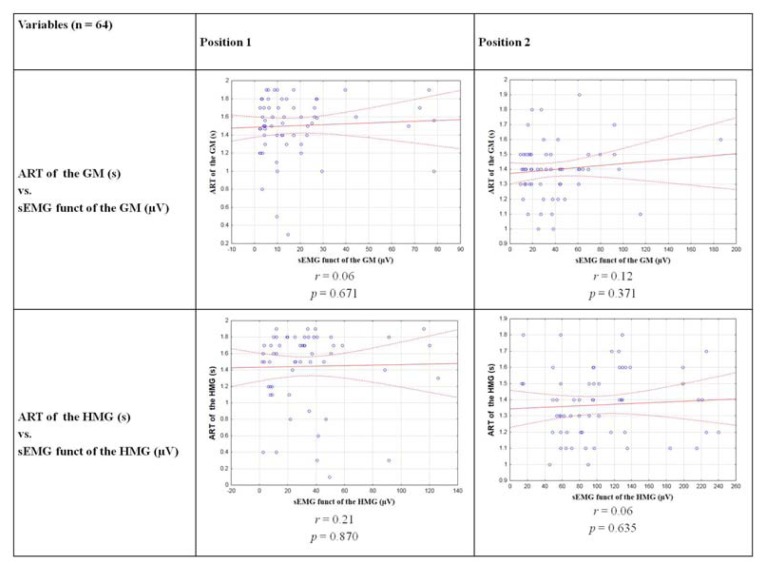
The correlations between the ART of the GM and HMG with their functional sEMG activity during hip extension in both positions

In the study population, no statistically significant correlation was found for all analysed variables. The Pearson correlation analysis showed statistically non-significant correlation between the results of ART of the GM or HMG and their functional sEMG activity (p<0.05). The correlations are presented in Figure 2.

### Differences in ART of the GM and HMG between males and females

There was no significant difference of ART between the results of female and male subjects in Position 1 and Position 2 (p>0.05). Also there was no significant difference of ART between both tested positions, in male as well as female subjects (p>0.05). In the female group, the relative onset differences between the GM and HMG in Position 1 and Position 2 were on average 0.11 s (range −1.2 – 1.4 ± 0.53 s) and 0.04 s (range −0.3 – 0.3 ± 0.16 s), respectively. In the female group, the relative onset differences between the GM and HMS in Position 1 and Position 2 were on average 0.00 s (range −0.7 – 1.3 ± 0.41 s) and 0.02 s (range −0.4 – 0.4 ± 0.17 s), respectively (Table 2).

## Discussion

Differences in ART of the GM and HMG during active hip extension are not statistically significant (our study is the first research including visually impaired athletes). It may indicate inter–individual differences in the hip joint extension pattern. It is interesting that, depending on the position, specific muscles of the same subjects are activated in a various pattern. To some extent, we obtained contradictory results to those obtained by Sakamoto et al. (2009), who presented that despite individual variability in healthy individuals, the order of muscle recruitment during PHE was similar for different modalities of therapeutic exercise (knee extension, knee flexion, lateral hip rotation and knee extension, lateral hip rotation and knee flexion), and in which the GM was consistently the last activated muscle. Kang et al. (2013) also reported a delayed GM sEMG onset relative to the HMG during PHE in the knee flexion exercise. However, in this study, subjects performed the exercise of PHE at the 0° hip abduction position as well as at the 15° and 30°. At the 0° hip abduction the HMG was activated earlier than the GM. In contrast, at the 15° and 30° hip abduction, the GM was activated as a first muscle.

Thus, the existence of a typical activation pattern remains controversial. We suggest that numerous factors can condition MP in adults, e.g.: lifestyle, environmental factors, individual habits or constitutional properties of the human body.

Lehman et al. (2004) assessed the sEMG activation of the GM, HMG, erector spinae (ES) and latissimus dorsi (LD) during PHE in fourteen asymptomatic subjects. The researchers registered a statistically significant delay of the GM in respect to the HMG. Other results were not statistically significant and indicated incoherent activation order of the HMG and ES.

Chance–Larsen et al. (2010) using sEMG device investigated whether specific training affected neuromuscular control of the biceps femoris (BF) and GM during PHE among twenty healthy volunteers. The intervention group performed a 10 min exercise with focus on proximal to distal muscle activation involving abdominal hollowing and active GM contraction prior to PHE. The control group undertook an exercise which included only PHE. Post exercise descriptive analysis indicated that the intervention exercise reduced the delay of the GM firing relative to BF. The results suggest that a short duration exercise intervention has the potential to alter the timing of activation of the GM relative to BF during PHE, yet this needs validation through future research.

In our study the low back pain, lower extremity injuries and pelvis asymmetry were exclusion criteria. However, Guimares et al. (2010) compared sEMG activity of the GM, semitendinosus (ST) and ES muscles between thirty asymptomatic and twenty individuals with low back pain during active PHE exercises. In their study no significant differences were found between the groups for any of the investigated muscles. Muscular activation patterns were similar for both groups for all studied muscles. For both groups, significant delays in the onset of the GM were observed.

Thus far, the relationship between gender in recruitment patterns of hip extension using sEMG examination remains unknown. We noticed some statistically significant differences in the ART of the GM between male and female subjects. However, our study is the first, thus requires further investigation and confirmation. Only Lewis and Sahrmann (2009) indicated that the muscle activation order during PHE was consistent in healthy women and demonstrated that muscle timing and activation amplitude and movement could be modified with verbal cues. It was concluded that the timing and amplitude of muscle activation, as well as the knee excursion, could be modified by verbal cues to use the GM to lift the leg while keeping the HMG relaxed.

Another important aspect is precise diagnostics and detection of MP. A method of palpation muscle activity assessment in the given MP may be found in the literature (Bruno et al., 2008; Janda et al., 1996). The authors tend to apply more precise and objective methods to detect the existing irregularities. The analyses performed by sEMG measurements indicate that delayed activation of the GM in respect to the HMG amounted to less than 0.1 in both tested positions. It appears that this time is insufficient for a reliable palpation assessment. That is why, an objective MP assessment may be provided by sEMG that may be widely applied in scientific research and clinical practice.

### Limitations of the study

There were several limitations in the present study that need to be considered. First, this study included a relatively small group of sixty four visually impaired individuals. It would be interesting to compare the obtained results with patients, e.g. after total hip replacement surgery, sacroiliac dysfunctions, musculoskeletal disorders or chronic low back pain. The second potential limitation of our study is that we did not use a higher sensitive multichannel sEMG, which would have registered more accurate variables, especially of ART. Another weakness of our study is that we collected sEMG data from only two selected muscles activated in the hip extension and this should upgrade the measurements to other muscles, e.g. erector spinae and abdominal muscles that maintain lumbar and pelvic stability. Finally, in this study, the sEMG registration was performed in only two positions in which the active hip extension was made. It may be valuable to include positions with different orientation of the pelvic tilt or to ensure some exercise position with a proper resistance, which could be developed to each muscle during the trials. We acknowledge the need to continue this research in a larger group of subjects and to perform some methodological improvements, but at the same time, we emphasize the pilot character of this study.

## Conclusions

Findings of the presented study suggest that in young visually impaired athletes, the ART of the GM was not consistently delayed with respect to the HMG during the active hip joint extension. A typical movement pattern during active hip extension in both tested positions as well as between males and females was not observed in our study. Perhaps inter–individual differences occurred in the activation order of the analysed muscles. Another significant finding of this study indicates that the initial average functional sEMG activity of the GM and HMG does not influence the recruitment time of these muscles, what might suggest that muscles bioelectrical properties in young healthy adults do not determine the muscle’s onset.

## Figures and Tables

**Table 1:** The resting and functional sEMG activity of the GM and HMG and their ART in both positions

Variables (n = 64)	GM		HMG		p*	p**	p***	p****
	Mean ± SD (min – max)		Mean ± SD (min – max)					
	Position 1	Position 2	Position 1	Position 2				
**sEMG rest (μV)**	2.23 *±* 1.27 (0.8 – 5.7)	3.02 ± 1.38 (0.9 – 6.4)	2.72 ± 1.38 (1.0 – 6.7)	4.05 ± 1.98 (0.9 – 8.9)	**0.0011**	**0.0000**	**0.0409**	**0.0011**
**sEMG funct (μV)**	17.42 ± 19.57 (2.3 – 78.5)	37.78 ± 31.38 (90 – 189.0)	33.30 *±* 30.99 (2.0 – 126.0)	102.81 ± 55.54 (13.5 – 240.0)	**0.0000**	**0.0000**	**0.0009**	**0.0000**
**ART (s)**	1.50 ± 0.33 (0.3 – 1.9)	1.40 ± 0.18 (1.0 – 1.9)	1.45 ± 0.45 (0.1 – 1.9)	1.37 ± 0.21 (1.0 – 1.8)	**0.0275**	0.2230	0.4163	0.4125

* GM – P1 vs P2

** HMG – P1 vs P2

*** P1 – GM vs. HMG

**** P2 – GM vs. HMG

Resting sEMG activity (sEMG rest); functional sEMG activity (sEMG funct); average recruitment time (ART); gluteus maximus (GM); hamstring muscle group (HMG). Comparing Position 1 and Position 2 for GM (p*); comparing Position 1 and Position 2 for HMG (p**); comparing GM and HMG in Position 1 (p***); comparing GM and HMG in Position 2 (p****)

**Table 2:** The differences of the average recruitment time between females and males in both positions

Variables (n = 64)	Female (n = 32)		Male (n = 32)		p*	p**	p***	p****
	Mean ± SD (min – max)		Mean ± SD (min – max)					
	Position 1	Position 2	Position 1	Position 2				
**ART GM – HMG (s)**	0.11 ± 0.53 (−1.2 – 1.4)	0.04 ± 0.16 (−0.3 – 0.3)	0.00 ± 0.41 (−0.7 – 1.3)	0.02 *±* 0.17 (−0.4 – 0.4)	0.4756	0.8557	0.3850	0.6447

Difference between average recruitment time of the gluteus maximus and hamstring muscle group (ART GM – HMG). Comparing Position 1 and Position 2 in females (p*); comparing Position 1 and Position 2 in males (p**); comparing males and females in Position 1 (p***); comparing males and females in Position 2 (p****)
